# Enhancement of Zyxin Promotes Skin Fibrosis by Regulating FAK/PI3K/AKT and TGF-β Signaling Pathways via Integrins

**DOI:** 10.7150/ijbs.77649

**Published:** 2023-04-29

**Authors:** Yan Huang, Han Zhao, Yuting Zhang, Yulong Tang, Xiangguang Shi, Shuai Jiang, Weilin Pu, Jing Liu, Yanyun Ma, Juiming Lin, Jinran Lin, Wenyu Wu, Yiyi Gong, Jiucun Wang, Qingmei Liu

**Affiliations:** 1 State Key Laboratory of Genetic Engineering, Collaborative Innovation Center for Genetics and Development, School of Life Sciences, and Human Phenome Institute, Fudan University, Shanghai, China.; 2 Nanjing Intellectual Property Protection Center, Nanjing, China.; 3 Division of Dermatology, Huashan Hospital, Fudan University, Shanghai Institute of Dermatology Shanghai, China.; 4 Institute for Six-sector Economy, Fudan University, Shanghai, China.; 5 Ministry of Education Key Laboratory of Contemporary Anthropology, School of Life Sciences, Fudan University, Shanghai, China.; 6 Department of Dermatology, Jing' an District Central Hospital, Shanghai, China.; 7 Research Unit of Dissecting the Population Genetics and Developing New Technologies for Treatment and Prevention of Skin Phenotypes and Dermatological Diseases (2019RU058), Chinese Academy of Medical Sciences, Beijing, China.

**Keywords:** Systemic sclerosis, Skin fibrosis, ECM, Zyxin, Focal adhesion, Integrins

## Abstract

Skin fibrosis is a common pathological manifestation in systemic sclerosis (SSc), keloid, and localized scleroderma (LS) characterized by fibroblast activation and excessive extracellular matrix (ECM) deposition. However, few effective drugs are available to treat skin fibrosis due to its unclear mechanisms. In our study, we reanalyzed skin RNA-sequencing data of Caucasian, African, and Hispanic SSc patients from the Gene Expression Omnibus (GEO) database. We found that the focal adhesion pathway was up-regulated and Zyxin appeared to be the primary focal adhesion protein involved in skin fibrosis, and we further verified its expression in Chinese skin tissues of several fibrotic diseases, including SSc, keloid, and LS. Moreover, we found Zyxin inhibition could significantly alleviate skin fibrosis using Zyxin knock-down and knock-out mice, nude mouse model and skin explants of human keloid. Double immunofluorescence staining showed that Zyxin was highly expressed in fibroblasts. Further analysis revealed pro-fibrotic gene expression and collagen production increased in Zyxin over-expressed fibroblasts, and decreased in Zyxin interfered SSc fibroblasts. In addition, transcriptome and cell culture analyses revealed Zyxin inhibition could effectively attenuate skin fibrosis by regulating the FAK/PI3K/AKT and TGF-β signaling pathways via integrins. These results suggest Zyxin appears a potential new therapeutic target for skin fibrosis.

## Introduction

Fibrosis is characterized by excessive deposition of extracellular matrix (ECM) and tissue remodeling, which leads to progressive tissue function loss and eventual organ failure 1. When ECM is deposited in skin along with activated fibroblasts, skin fibrosis will occur 2. Systemic sclerosis (SSc), localized scleroderma (LS) and keloid are typical skin fibrotic diseases 3. SSc is an immune-mediated rheumatic disease characterized by cutaneous fibrosis and internal organ involvement 4, whereas LS is characterized by localized skin thickening usually without severe systemic symptoms or Raynaud phenomenon. In general, LS is often self-limited and with a good prognosis 5, 6. Keloid is a fibro-proliferative disease reflecting an abnormal process of wound healing. It may arise after deep cutaneous injury from causes such as trauma, burns, and surgery 7, 8. Several approaches have been applied to treat fibrotic diseases, including methotrexate, mycophenolate mofetil, penicillamine, intralesional corticosteroid injections, and surgery 9, 10. Unfortunately, the treatment of skin fibrosis still poses a significant challenge due to the limited therapeutic effects, potentially hazardous side effects, and varying degrees of recurrence. A deeper understanding of the underlying mechanisms is crucial for developing effective anti-fibrotic treatments.

Fibroblast could synthesize collagen and remodels the ECM and is considered to be the predominant effector cell type involves in skin fibrosis 11, 12. Multiple signaling pathways have been reported to participate in the progression of fibroblast activation and skin fibrosis. Recently, the role of focal adhesion in fibrosis has attracted attention. Focal adhesion is a macromolecular complex formed by focal adhesion proteins such as zyxin, vinculin, paxillin, talin, α-actinin, kindlin, tensin, and focal adhesion kinase (FAK), serves as a mechanical connection to the ECM and acts as a biochemical signal hub to concentrate and direct numerous signal proteins and plays an important role in many biological processes 13, 14.

Zyxin is one of the key components of focal adhesion, and is essential for cell activities, such as cell adhesion, cell migration, and cell proliferation 15, 16. Disruption or disorder of Zyxin may lead to the dysfunction of the focal adhesion complex, further lead to multiple diseases, including cancers and autoimmune diseases 17. However, the role of Zyxin in the development of skin fibrosis remains elusive. Herein, our study found the increased expression of Zyxin in fibrotic diseases' lesion skins, identified the role of Zyxin in skin fibrosis using *in vivo* models and keloid skin explants, and revealed the molecular mechanism of Zyxin in skin fibrosis through *in vitro* studies.

## Results

### RNA-seq reveals upregulated focal adhesion signaling pathway and enhanced Zyxin in fibrotic skin tissues

**Figure 1:**
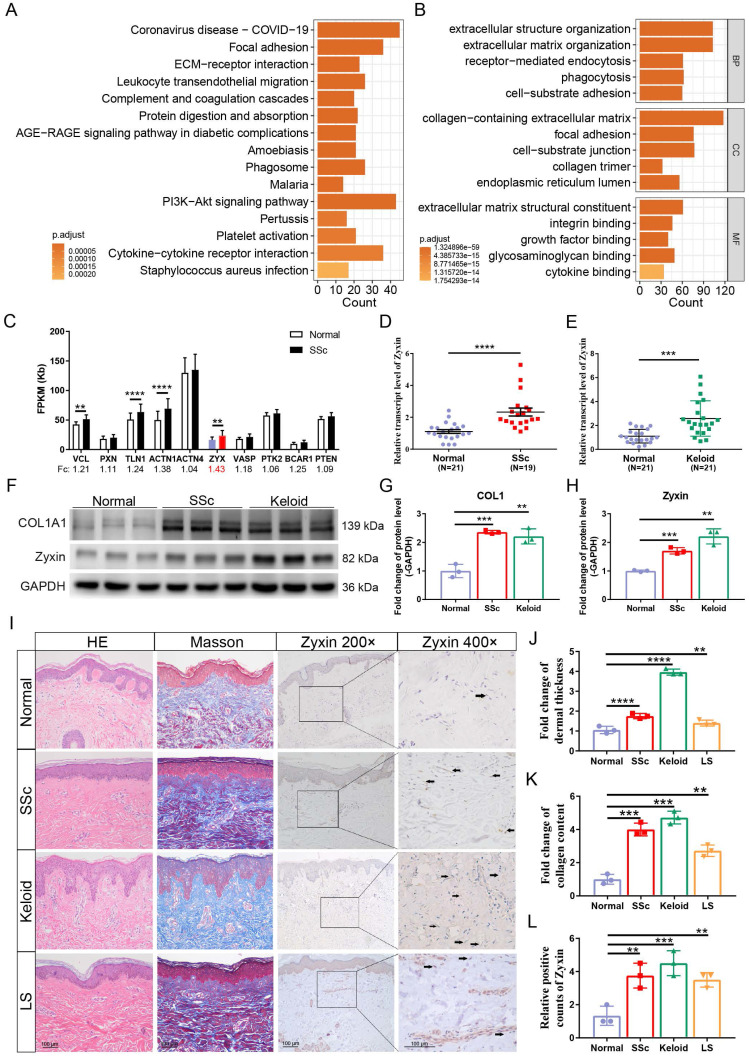
** RNA-seq reveals changed signaling pathways and enhanced Zyxin expression in SSc, keloid, and LS.** (A, B) KEGG and GO enrichment analysis of DEGs between normal and SSc from GEO database. (C) FPKM (Fragments Per Kilobase per Million) of focal adhesion proteins in the focal adhesion signaling pathway. (D) Relative transcript levels of Zyxin in normal (n=21) and SSc (n=19) were measured by Real-time PCR. (E) Relative transcript levels of Zyxin in normal (n=21) and keloid (n=21) were measured by Real-time PCR. (F) Relative protein levels of COL1A1 and Zyxin in the skin tissue of normal, SSc, and keloid. (G, H) Semi-quantification of the Western blot results by ImageJ. (I) HE, Masson trichrome, and IHC staining in normal, SSc, keloid, and LS. (J, K) Fold change of dermal thickness and collagen content in SSc, keloid, and LS determined from photomicrographs using ImageJ. (L) Relative Zyxin positive counts of different groups. Data are presented as means±SD in two groups and compared by *t*-test. *P<0.05, **P<0.01, ***P<0.001, ****P<0.0001.

To explore the genes involved in the pathogenesis of skin fibrosis, we performed the transcriptome analysis using the RNA-seq data of 33 normal and 48 SSc skin tissues consisting of Caucasian, African, and Hispanic people from the Gene Expression Omnibus (GEO) database (GSE130955) 18. We identified 2668 differentially expressed genes (DEGs), and 76 DEGs were enriched in the focal adhesion pathway, highlighting its role in skin fibrosis (Figures 1A, B, and Table S1). Then, we calculated the expression levels of focal adhesion proteins in the enriched focal adhesion pathway and found that *Zyxin* showed the highest fold change (FC = 1.43) between fibrotic and normal skin tissues, suggesting that *Zyxin* is significantly altered in fibrotic skin tissues (Figure 1C).

To verify this finding, we examined *Zyxin* expression in an independent sample group from the Chinese population. We determined the expression of *Zyxin* in the skin tissues from SSc (n = 19) and keloid (n = 21) patients and normal samples (n = 21) and found a significant up-regulation of *Zyxin* in both SSc (2.32 ± 0.24 vs 1.11 ± 0.11, P < 0.01) (Figure 1D) and keloid patients (2.58 ± 0.32 vs 1.10 ± 0.12, P < 0.01) (Figure 1E). Meanwhile, the protein levels of Zyxin and collagen I were also elevated in the skin tissues of SSc and keloid patients (Figures 1F-H). Moreover, skin tissue samples from LS, another skin fibrotic disease, were then added for further histological analysis. HE and Masson's staining highlighted significant skin thickening and collagen production in SSc, keloid, and LS skin tissues (Figures 1I-K). In particular, the IHC staining also showed more Zyxin-positive cells in the dermis of SSc, keloid, and LS patients than in normal controls (Figures 1L). These results indicated that Zyxin expression was increased in the skins of SSc, keloid, and LS patients, hinting at the important role of Zyxin in skin fibrosis.

### Loss of Zyxin alleviates skin fibrosis in Bleomycin (BLM) model mice

**Figure 2:**
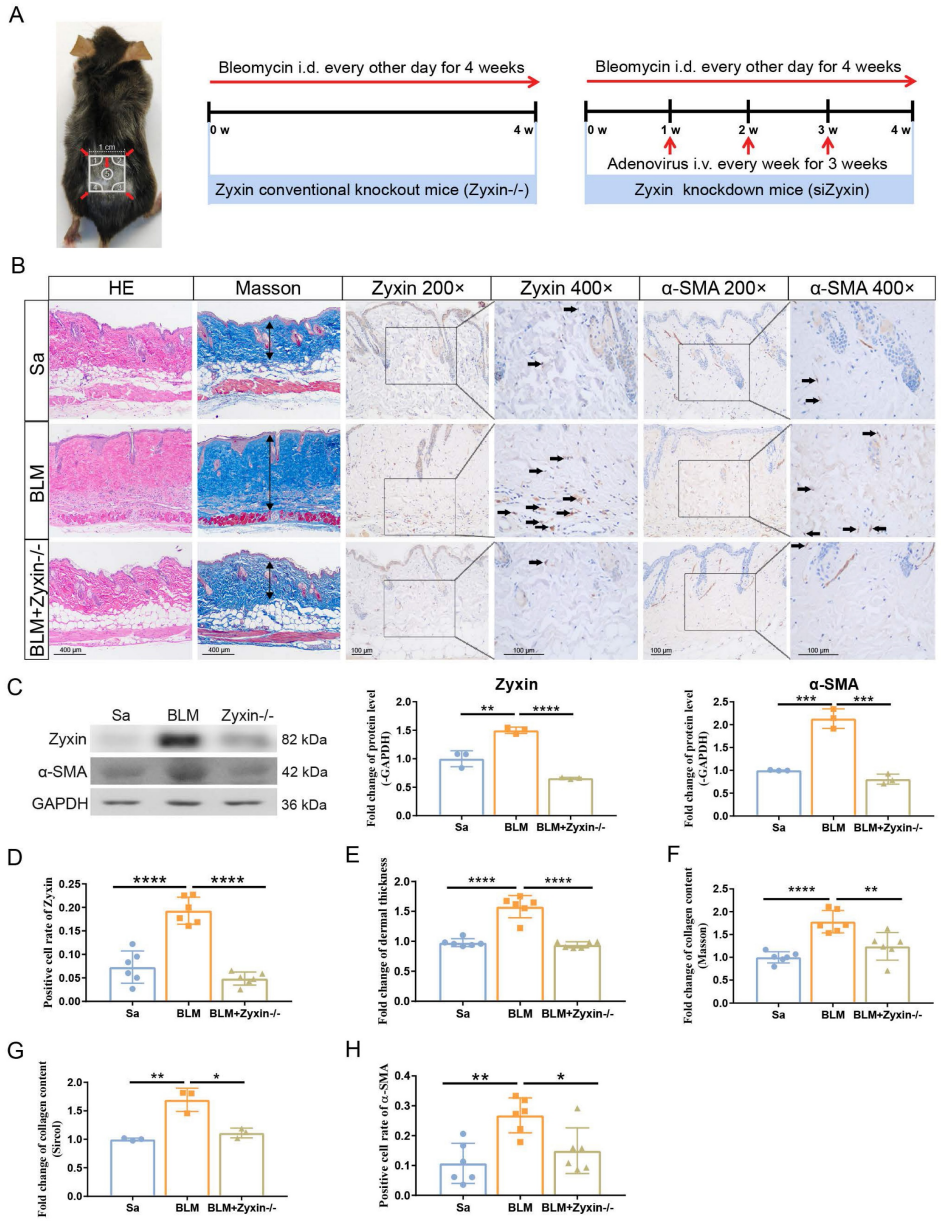
** Knockout Zyxin alleviated skin fibrosis in BLM-induced model mice.** (A) The protocol of the experimental mouse model. (B) Sections were stained with HE, Masson's trichrome, and IHC staining of different treatment groups among Zyxin knockout mice. The arrow indicated Zyxin-positive cells. (C) Relative protein levels of α-SMA and Zyxin in mouse skin were measured by Western blotting. (D) The positive cell rate of Zyxin in different treatment groups. (E) Dermal thickness of mouse skin in different groups was calculated. (F, G) Collagen content of mouse skin in different groups was measured by ImageJ and Sircol collagen kit. (H) The positive cell rate of α-SMA in different treatment groups. Data are presented as mean±SD in two groups and compared by *t*-test. *P<0.05, **P<0.01, ***P<0.001, ****P<0.0001.

To further clarify the role of Zyxin in skin fibrosis, we compared the BLM-induced skin fibrosis in Zyxin knockout and knockdown mice with wild-type (WT) mice (Figure 2A). As revealed by IHC and western blotting, the protein level of Zyxin was upregulated (4.6-fold) in the BLM-treated WT mice and significantly downregulated (7.0-fold) in the BLM-treated Zyxin^-/-^ mice (Figures 2B-D). Moreover, a histological analysis was conducted to explore the remission effect of decreased Zyxin on skin fibrosis. Compared with the BLM-treated WT mice, BLM-treated Zyxin^-/-^ mice exhibited less dermal thickness and collagen content (Figures 2E and F). Consistently, the Sircol assay also showed a decreased collagen content in Zyxin^-/-^ mice compared to WT mice with the treatment of BLM (Figure 2G).

In addition, the protein level of α-SMA and the number of α-SMA positive myofibroblasts in lesion skin of Zyxin^-/-^ mice were both significantly decreased in comparison with that of BLM treated WT mice (Figure 2C and H). Furthermore, we performed shRNA-mediated knock-down experiments to further verify the role of Zyxin in skin fibrosis. We found the Zyxin knockdown mice showed very similar phenotypes compared to the Zyxin^-/-^ mice, including less dermal thickening and decreased collagen content (Figures S1A-G). Taken together, these results suggested that Zyxin played an essential role in the development of skin fibrosis and inhibition of Zyxin might protect against skin fibrosis.

### Interfering Zyxin attenuates skin fibrosis in keloid skin explants and nude mouse model

**Figure 3:**
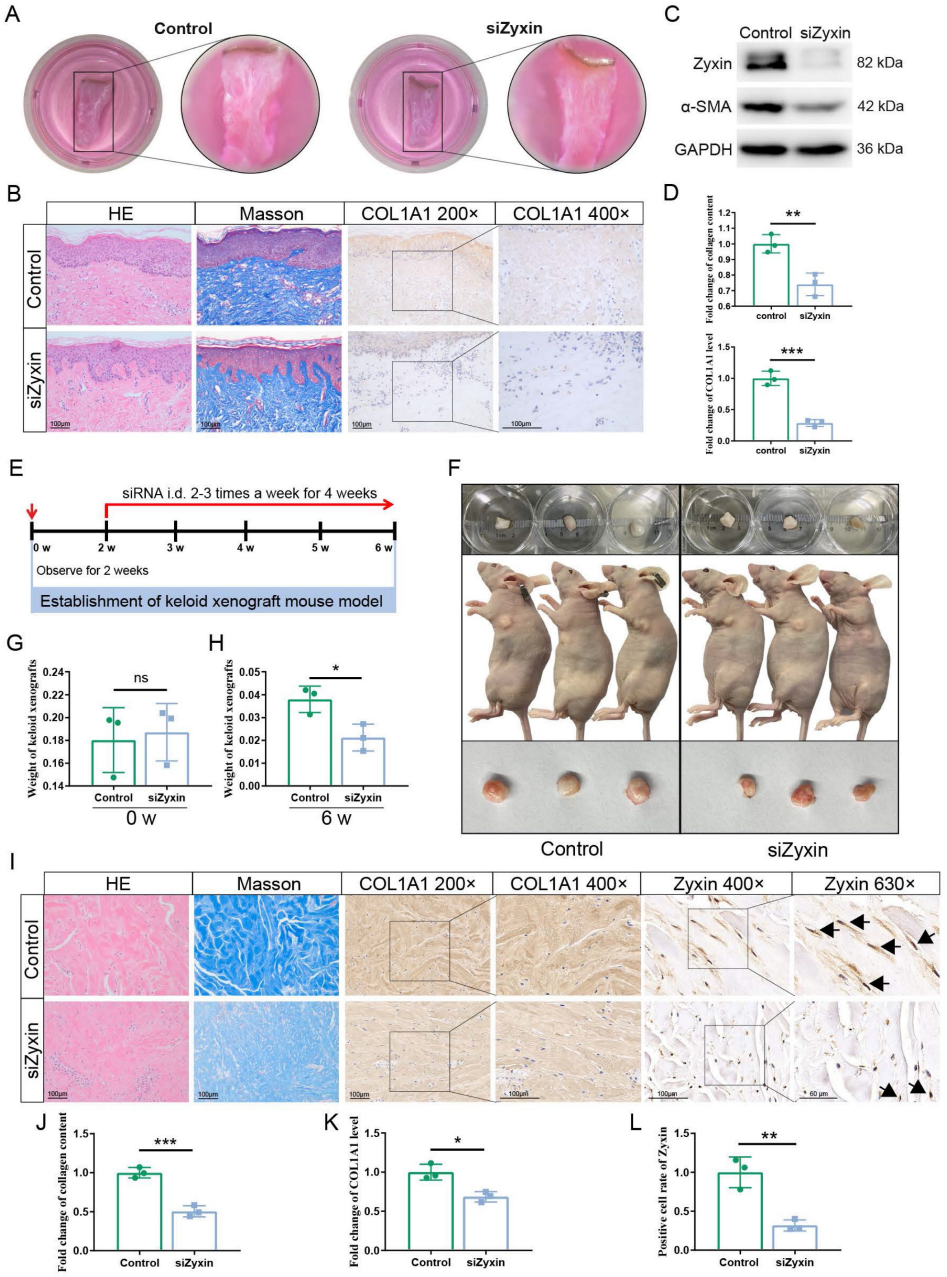
** Interfering Zyxin attenuates skin fibrosis in keloid skin explants and nude model mice.** (A) Treatment status of keloid skin explants. (B) Sections were stained with HE, Masson's trichrome, and immunohistochemistry staining of different treatment groups. (C) Relative protein levels of α-SMA and Zyxin in keloid skin explants were measured by Western blotting. (D) Collagen content of different treatment groups showed in Masson's staining was measured by ImageJ (Upper). Protein level of COL1A1 in immunohistochemistry staining among different treatment groups measured by ImageJ (Lower). (E) The protocol of the keloid xenograft nude mouse model. (F) The size and status of keloid tissues before embedding in nude mice (Time: 0 w, upper). The size and location of keloid tissues in nude mice (Time: 6 w, middle). The size of keloid tissues harvested in the 6^th^ week (Time: 6 w, lower). (G, H) The weights of keloid tissues before embedding and after harvesting from nude mice in the 0^th^ and 6^th^ week. (I) The HE, Masson, and IHC staining of keloid tissues at the end of different treatments and their quantitative results (J, K, L). Data are presented as mean±SD in two groups and compared by *t*-test. *P<0.05, **P<0.01, ***P<0.001, ****P<0.0001.

In addition to BLM-induced skin fibrosis mouse model, skin explants have very similar characteristics with live skin tissues in terms of structure and physiology, and would be an ideal model to verify the role of Zyxin in skin fibrosis. Therefore, we used the keloid skin explants and interfered Zyxin with siRNA subcutaneously injection (Figure 3A). As revealed by western blotting, the protein levels of Zyxin and α-SMA were significantly downregulated after interfering Zyxin in keloid skin explants (Figures 3B and C). Furthermore, the siZyxin explants exhibited a significant decrease of dermal collagen content in Masson's staining (Figures 3B and D). Consistently, the IHC staining showed that COL1A1 diffused in keloid skin dermis was significantly decreased in siZyxin group (Figures 3B and D). These results indicated that interfering Zyxin could alleviate skin fibrosis in keloid skin explants - a biological system explanted from the human body.

In human keloid xenograft nude mouse model, we found that the weight of keloid tissue in the Zyxin siRNA group was 0.021 ± 0.003g, which is significantly decreased than the PBS control group (0.038 ± 0.003g, P < 0.05) (Figures 3E-H). As revealed by IHC and Masson's staining, the siZyxin keloid tissue exhibited a significant decrease in Zyxin expression and dermal collagen content (Figures 3I-L). These results indicated that interfering Zyxin could alleviate skin fibrosis in keloid xenograft nude mouse model, further validating the role of Zyxin in skin fibrosis.

### Zyxin is highly expressed in fibrotic skin fibroblasts and promotes the activation of skin fibroblasts

**Figure 4:**
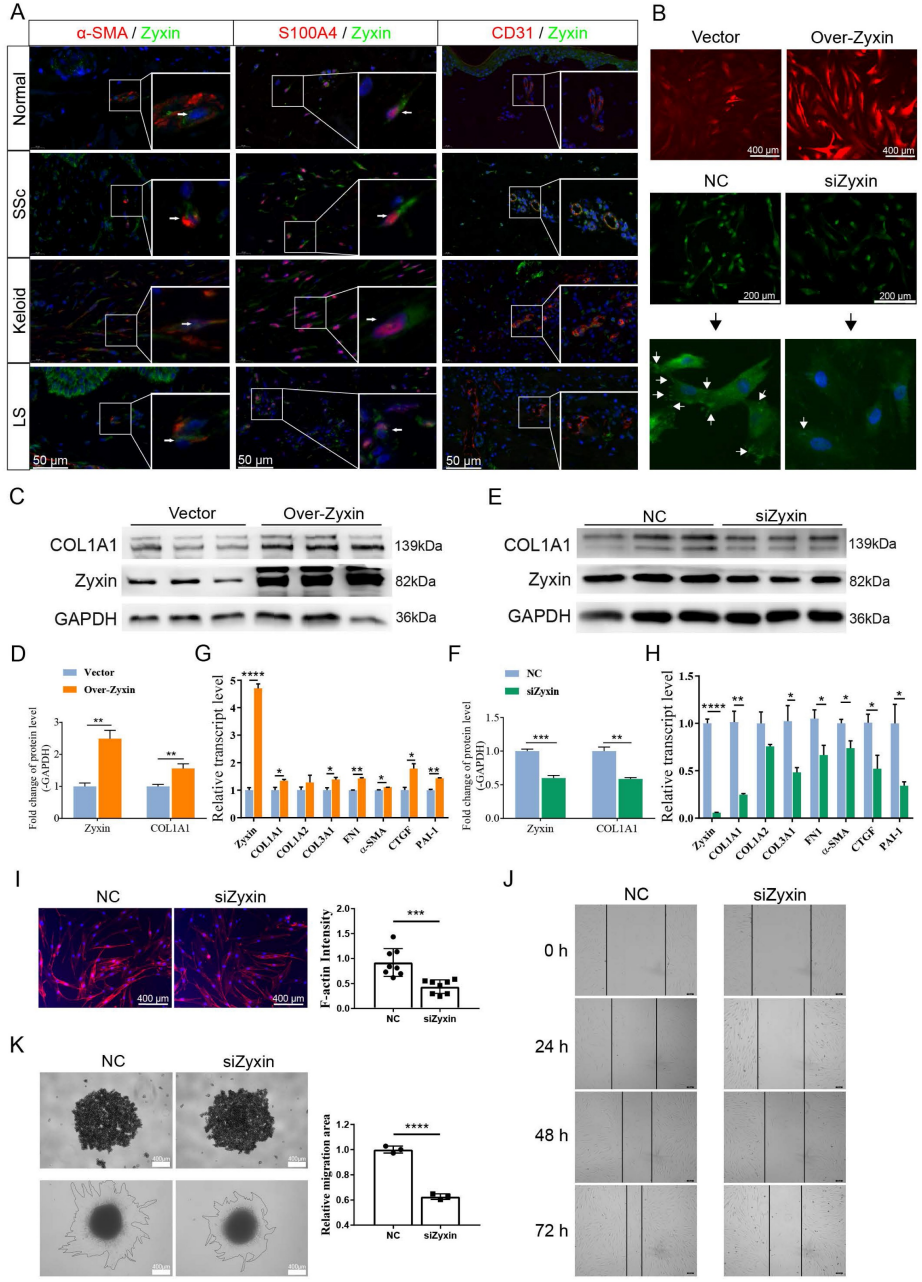
** High Zyxin expression level promotes activation and migration of fibroblasts.** (A) Double immunofluorescence staining in normal, SSc, keloid, and LS sections. Arrows indicate the positive cells. (B) Immunofluorescence staining of Zyxin in Zyxin over-expressed and knock-down fibroblasts. (C-F) Relative protein levels of COL1A1 and Zyxin in different treatment groups. Semi-quantification of the Western blot results by ImageJ. (G, H) Expression level of fibrotic genes was measured by Real-time PCR. (I) F-actin through the phalloidin staining on SSc primary fibroblasts. (J) Scratch assay was performed on primary fibroblast treated with or without Zyxin siRNA. (K) 3D spheroid model migration test was applied to measure the migration of SSc primary fibroblasts. Data are presented as means±SD in two groups and compared by *t*-test. *P<0.05, **P<0.01, ***P<0.001, ****P<0.0001.

To identify the Zyxin expression programs in different cell types, we performed a double immunofluorescence staining and found that Zyxin was highly expressed in fibroblasts (α-SMA or S100A4 marked) rather than endothelial cells (CD31 marked) in fibrotic skin tissues (Figure 4A). Therefore, we over-expressed Zyxin in HFF-1 cell line and knocked down Zyxin in SSc primary fibroblasts to explore the role of Zyxin in fibroblasts (Figure 4B). Expectedly, the protein levels of Zyxin and collagen I were increased in Zyxin over-expressed HFF-1 cells, and decreased in SSc primary fibroblasts after Zyxin knock-down treatment (Figures 4C-F). Moreover, fibrosis-related genes, including *COL1A1, COL1A2, COL3A1, FN1, α-SMA, CTGF,* and* PAI-1* were up-regulated in Zyxin over-expressed HFF-1 cells while down-regulated after interfering Zyxin (Figures 4G and H).

Previous studies have found that activated fibroblasts can sense mechanical stress, migrate to the reticular dermis with the aid of F-actin protein, cause skin fibrosis. Therefore, we assessed the density of F-actin on SSc primary fibroblasts after inhibiting Zyxin, and found a significant decrease of F-actin after Zyxin knocked-down treatment (Figure 4I). Additionally, scratch assay and 3D spheroid model migration (Matrigel) test revealed that Zyxin knockdown significantly reduced cell migration (Figures 4J and K). Moreover, we used RTCA in fibroblasts to investigate if Zyxin might affect the proliferation of fibroblasts, but no significant difference was found between the Zyxin knockdown and control groups (Figures S2A and B). These findings suggested that Zyxin played a pro-fibrotic role in fibroblasts and contributed to skin fibrosis.

### Zyxin siRNA transfected fibroblasts had enhanced the FAK/PI3K/AKT and TGF-β signaling pathways

**Figure 5:**
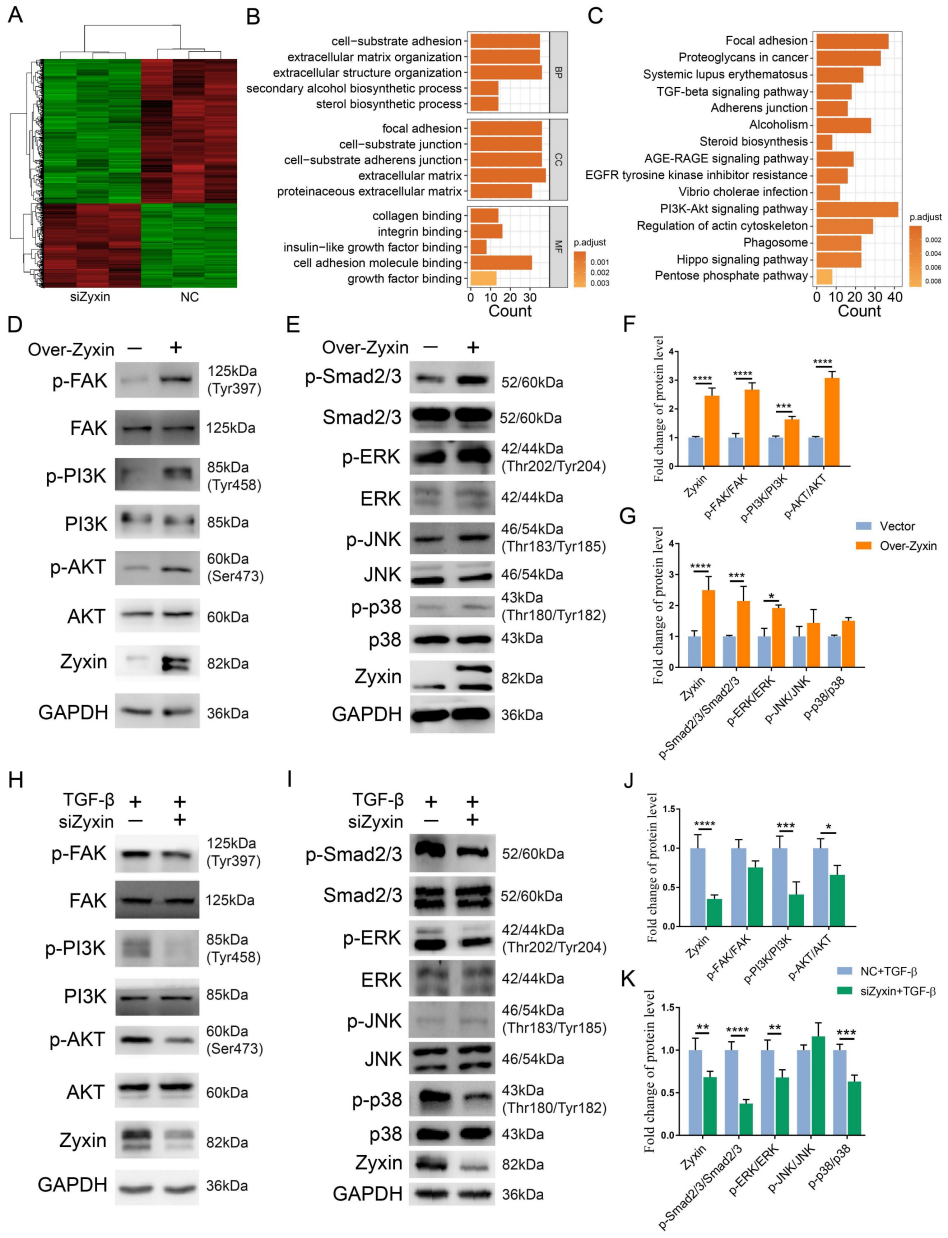
** Focal adhesion and TGF-β signaling pathways changed in Zyxin interference fibroblasts.** (A) Heatmap of all the DEGs between NC and siZyxin treatment groups. (B) GO analysis of DEGs. Different colors indicate different levels of -log(P-value). (C) KEGG pathway analysis of DEGs. (D, E) Levels of phosphorylated and total FAK, PI3K, and AKT were assessed in fibroblasts with or without Zyxin over-expression and knock-down. (F, G) Semi-quantification of the Western blot results by ImageJ. (H, I) Levels of phosphorylated and total Smad2/3, ERK, JNK, or p38 were assessed in fibroblasts with or without Zyxin over-expression and knock-down. (J, K) Semi-quantification of the Western blot results by ImageJ. Data are presented as means±SD in two groups and compared by *t*-test. *P<0.05, **P<0.01, ***P<0.001, ****P<0.0001.

To clarify the mechanism of Zyxin in promoting skin fibrosis, we performed RNA sequencing on primary fibroblast from SSc patients with or without Zyxin interference. The DEGs were enriched in pathways related to focal adhesion, PI3K-AKT, and TGF-β signaling pathways (Figures 5A-C), which were further supported by IHC staining (Figure S3).

As a result, we assumed that the prevention of fibrosis by Zyxin interference may act through the FAK/PI3K/AKT and TGF-β pathways. To validate this hypothesis, we performed over-expression of Zyxin in HFF-1 cells, and found Zyxin over-expressed fibroblasts displayed higher level of Zyxin expression and protein phosphorylation level in the FAK/PI3K/AKT and TGF-β pathways as compared to the vector group (Figures 5D, E). In addition, these fibroblasts showed a significant increase in the ratios of p-FAK/FAK, p-PI3K/PI3K, p-AKT/AKT, p-Smad2-3/Smad2-3 and p-ERK/ERK (Fc = 2.67, 1.64, 3.08, 2.15, 1.91, respectively) (Figures 5F and G), while the ratios of p-JNK/JNK and p-p38/p38 had increasing trends without statistical significance (P = 0.37, 0.22) (Figure 5G). In contrast, after interfering Zyxin in HFF-1 cells, we detected corresponding protein phosphorylation levels through western blot, but only saw slight changes in the siZyxin group compared to those in the NC group (Figures S4A-J). However, interfering Zyxin dramatically decreased the phosphorylation level of PI3K, AKT, Smad2/3, ERK, and p38 (Fc = 2.44, 1.51, 2.68, 1.47, 1.58) following the fibrotic treatment with TGF-β (Figures 5H-K). Collectively, these findings suggest that Zyxin may regulate the FAK/PI3K/AKT and TGF-β pathways in fibroblasts.

### Decreased Zyxin attenuated the fibrosis procedure through the FAK/PI3K/AKT and TGF-β signaling pathways via integrins

**Figure 6:**
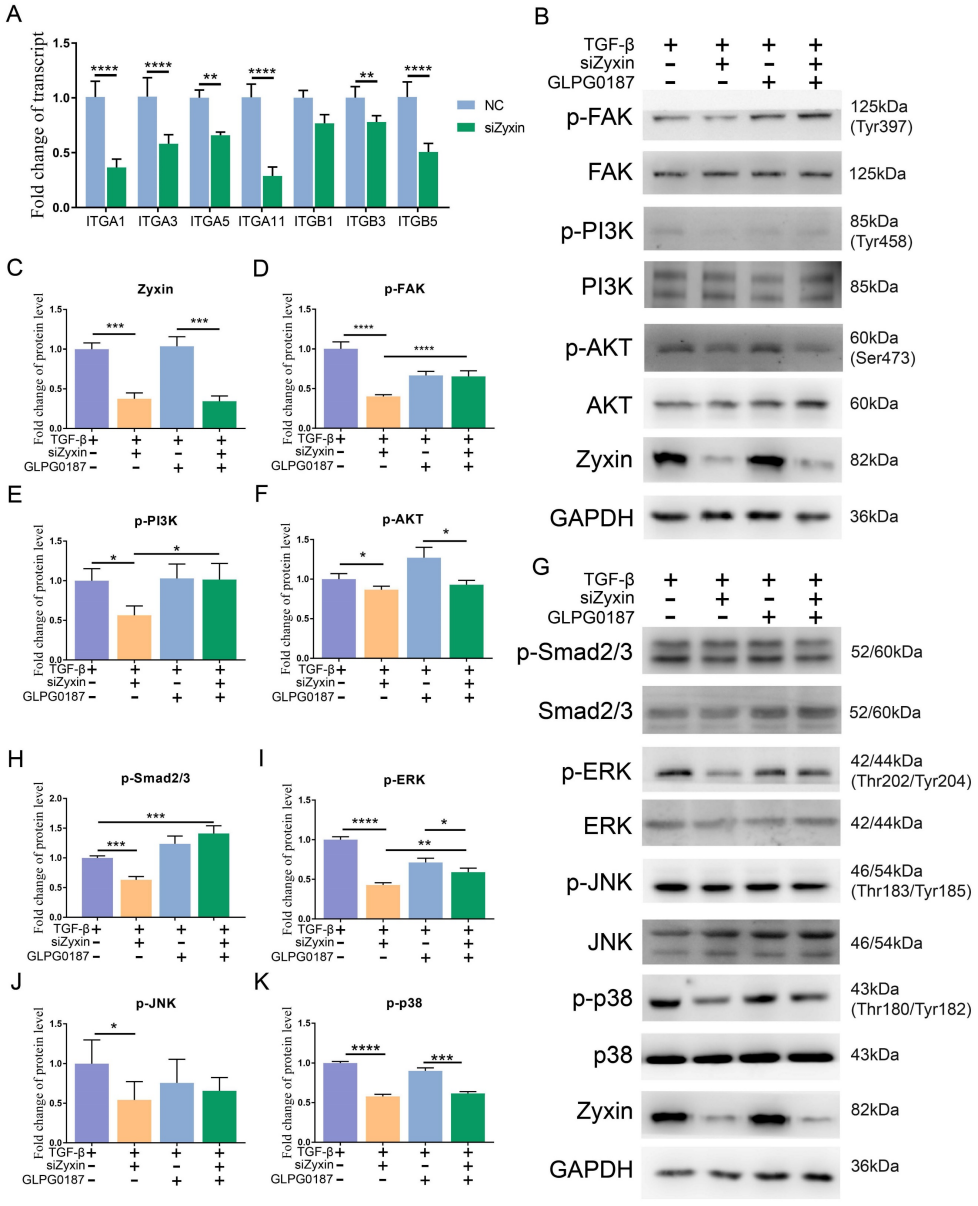
** The role of integrins in mediating Focal adhesion and TGF-β signaling pathways regulated by Zyxin in fibroblasts.** (A) Relative transcript levels of integrins in primary fibroblasts separated from SSc patients with or without pretreatment of Zyxin siRNA were measured by Real-time PCR. (B) Levels of phosphorylated and total FAK, PI3K, or AKT were assessed in fibroblasts by Western blotting. (C-F) Semi-quantification of the Western blot results by ImageJ. (G) Levels of phosphorylated and total Smad2/3, ERK, JNK, or p38 were assessed in fibroblasts by Western blot. (H-K) Semi-quantification of the Western blot results by ImageJ. Data are presented as means±SD in two groups and compared by *t*-test. *P<0.05, **P<0.01, ***P<0.001, ****P<0.0001.

Integrins are located upstream of the FAK/PI3K/AKT pathway and play an important role in the interaction between focal adhesions and the extracellular matrix; the activation of the TGF-β signaling pathway also depends on integrins. Based on our RNA-seq data, integrins were generally decreased in Zyxin interfered SSc primary fibroblasts (Table S2), qPCR on several common integrin family members also confirmed the changes (Figure 6A). In light of this, we proposed that Zyxin may regulate the FAK/PI3K/AKT and TGF-β pathways by means of integrins. We then applied the pan integrin antagonist GLPG0187 in the fibroblasts. Without the pre-treatment of TGF-β, as previously noted, there were no significant changes in the phosphorylation levels of these two signaling pathways (Figures S4A-J). In contrast, after the TGF-β treatment, the phosphorylation levels of the FAK/PI3K/AKT and TGF-β pathways were significantly decreased in the fibroblasts with interfered Zyxin. However, this alteration was not seen in the fibroblasts pretreated with GLPG0187 prior to interfering Zyxin (Figures 6B-K). Moreover, the *in vitro* cell experiments showed that integrin blocker GLPG0187 treatment did not influence the expression of Zyxin (Figures S5A, B). Due to the effects of GLPG0187 in decreasing the change of the phosphorylation levels of FAK, AKT, PI3K, ERK, JNK, and p38 in Zyxin interfered fibroblasts, we believed that interfering Zyxin could influence these pathways by down-regulating integrin families. These results reveal that Zyxin may regulate the expression of integrins to affect the activation of the FAK/PI3K/AKT and TGF-β pathways.

## Discussion

In the present study, we analyzed public RNA sequencing data of SSc skin in Caucasian, African, and Hispanic people, and identified the focal adhesion signaling pathway as the most enriched pathway. The main effector of this pathway was focal adhesion consisting of various focal adhesion proteins. It functions as a biochemical signaling hub that can transduce various signals and as a mechanical linkage to the ECM that senses mechanical stress 19-21. Further analysis revealed that, based on the RNA-seq data, Zyxin in SSc skin appeared to have the biggest fold change in expression when compared to other focal adhesion proteins. This change was further validated in an independent Chinese sample group consisting of SSc, keloid, and LS patients. The high expression level of Zyxin in different ethnic and fibrotic diseases highlights its potential function in skin fibrosis.

Indeed, Zyxin knockdown and knockout mice showed significant resistance against skin fibrosis induced by BLM, which is compelling evidence for Zyxin's critical role in skin fibrosis. The bleomycin model of scleroderma is characterized by infiltration of mononuclear cells into the lesional skin, and thickening of the dermis that is maintained for at least 6 weeks 22. Yamamoto and Chandrasekaran have found that this model simulates not only skin fibrosis but also scleroderma pulmonary fibrosis 22, 23. Although this model cannot completely simulate multiple organ lesions occurring in SSc patients, it still a commonly used mouse model for skin fibrosis 24-26. To better recapitalize the pathogenesis of other skin fibrotic diseases, like keloid, we constructed a keloid nude mouse model and skin explant model, and found that Zyxin inhibition could alleviate skin fibrosis of keloid. The results from the studies of both *in vivo* animal model and *ex vivo* human tissue explant support the important role of Zyxin in skin fibrosis and make it possible to develop treatments for skin fibrosis by employing small molecule inhibitors and small interfering RNA of Zyxin.

In the fibrotic dermis, fibroblast is recognized as a central player and its activation contributes to ECM production 27, 28. In our study, double immunofluorescence staining showed Zyxin was highly expressed in fibrotic skin fibroblasts. According to previous studies, Zyxin plays an essential role in cell adhesion, cell migration, and tissue response to mechanical stress 29-31. However, the role of Zyxin in fibrotic skin fibroblasts remains unclear. In this study, we revealed that the expression level of Zyxin was positively correlated with the production of collagen in fibroblasts. Furthermore, fibroblast migration is thought to play a role in the local progression of fibrosis, including cell recruitment to the fibrotic areas and pathogenic cell migration to healthy tissue 32. Our results also revealed that Zyxin could influence the migration capacity of fibroblasts, which provided strong evidence of the pro-fibrosis effect of Zyixn through fibroblasts.

Up-regulation of TGF-β in fibrotic diseases leads to the overproduction of collagen and ECM by activating fibroblasts 33. Previous studies reported that the activation of the TGF-β signaling pathway enhanced the expression of integrins, and integrins could affect the activation of the TGF-β pathway in turn 34, 35. For instance, a small-molecule inhibitor of the α_v_β_1_ integrin was shown to inhibit the activation of TGF-β in primary lung and liver fibroblasts 36. In our study, we also found that interfering Zyxin could reduce the expression of integrins and further inhibit the activation of the TGF-β signaling pathway.

Focal adhesion pathway was enriched both in SSc patient skins and in SSc fibroblasts, suggesting it plays an essential role in skin fibrosis. Focal adhesion is linked to mucins in the ECM via integrins, then the biological functions of focal adhesion, like signaling transduction, rely on the activation of integrins 37. Itga6/FAK/PI3K/AKT signaling cascade pathway has been reported to be an important signaling pathway in silica-induced pulmonary fibrosis 38. Consistent with our results, the FAK/PI3K/AKT signaling pathway was inactivated after interfering Zyxin for its down-regulation of integrins. In addition to Zyxin, several other focal adhesion proteins have also been reported to be related to fibrosis, such as FAK, vinculin, and kindlin-2 39-41. The role of those focal adhesion proteins in skin fibrosis requires further exploration in the future.

Furthermore, the inhibition of interfering Zyxin on the FAK/PI3K/AKT and TGF-β signaling pathways occurred in TGF-β stimulated fibroblasts, while interfering Zyxin had little effect on these signaling pathways of normal fibroblasts without TGF-β stimulation, indicating that Zyxin is a good therapeutic target for fibrosis. Although our study clearly showed that Zyxin regulated the FAK/PI3K/AKT and TGF-β signaling pathways through integrins in fibrotic fibroblasts, future experiments are still needed on the role of specific integrins in these signaling pathways.

In summary, our study characterized the function and mechanism of Zyxin in fibroblast activation and skin fibrosis. We reported the up-regulation of Zyxin in the fibroblasts of lesional skins from patients with fibrotic diseases and demonstrated antifibrotic effects of Zyxin on *in vivo* Zyxin knockdown and knockout mice, keloid xenograft mouse model, as well as *ex vivo* keloid skin explants. The results also presented clear evidence that Zyxin was involved in the fibrogenic process and fibroblast activation by the FAK/PI3K/AKT and TGF-β signaling pathways via integrins. In conclusion, our findings support the extension of the targeting of the FAK/PI3K/AKT and TGF-β signaling pathways as an anti-fibrotic strategy in fibrosis skin, and Zyxin appears to be a potent therapeutic target against skin fibrosis.

## Experimental Procedures

### Patients and normal subjects

Keloid, SSc patients, and normal controls were recruited from Shanghai Huashan Hospital. Patients with SSc were diagnosed and classified according to the 2013 American College of Rheumatology (ACR)/ European League of Rheumatology (EULAR) classification criteria. Patients with keloid were diagnosed according to the classification and evaluation criteria of the JSW Scar Scale (JSS)2015. Age- and gender-matched healthy Chinese individuals were enrolled as normal controls. The study was approved by Fudan University and written informed consent was obtained from all subjects.

### Histological analysis

Immunohistochemistry was performed on paraformaldehyde-fixed paraffin sections. Skin sections were dewaxed, microwaved in citrate-buffered saline, and incubated with antibodies of pPI3K (1:100, Cell Signaling Technology, USA), pAKT (1:100, Cell Signaling Technology, USA), pFAK(1:50, Cell Signaling Technology, USA) at 4°C overnight. After washing, sections were labeled with horseradish peroxidase (HRP)-conjugated secondary antibodies (servicebio, China) for 12 h at room temperature and visualized with DAB peroxidase substrate (servicebio, China). Finally, sections were counterstained with haematoxylin (servicebio, China). All the images were observed and photographed using a microscope (Nikon, Japan).

### Immunofluorescence analysis

Dual immunofluorescence staining experiments for α-SMA/Zyxin and s100a4 /Zyxin were performed on paraffin sections. The sections were sealed with bovine serum albumin and incubated overnight at 4°C with the respective primary antibody, including α-SMA (1:250, Abcam, UK), s100a4(1:100, Abcam, UK), and Zyxin (1:500, Abcam, UK). Then, the sections were incubated with a second antibody at room temperature for 1 h. After the second antibody was removed, the nuclei were counterstained with 4-amino-6-diamino-2-phenyl indole (DAPI, Beyotime Biotechnology, China). Fluorescence confocal images were captured using an LSM 5 Pascal Laser Scanning Microscope (Zeiss, Germany).

### RNA isolation, reverse transcription, and real-time RT-PCR

Total RNA samples were extracted from skin biopsies using TRIzol (Invitrogen, USA) according to the manufacturer's instructions. Complementary DNA (cDNA) was synthesized using High-Capacity cDNA Reverse Transcription Kit (Applied Biosystems, USA). Real-time PCR was performed with SYBR Green I PCR Kit (TaKaRa, Japan) and analyzed with an ABI Prism 7900 Detector System (Applied Biosystems, USA). The Real-time RT-PCR primers are listed in Table S3 and the data were analyzed with SDS 2.3 software (Applied Biosystems, USA).

### Establishment of skin fibrosis mouse model

C57BL/6 wild type (WT) mice were purchased from Shanghai Laboratory Animal Center (Chinese Academy of Sciences, China) and Zyxin conventional knockout (Zyxin^-/-^) mice were purchased from Cyagen (KOCMP-00390-Zyx). All mice were bred and housed in the Animal Center of Fudan University. Seven-week-old female C57BL/6 mice (were randomly divided into three groups: Control, BLM (Nippon Kayaku, Tokyo, Japan), and siZyxin group. In the BLM and siZyxin group, mice were subcutaneously injected with 100 μL BLM (200 μg/mL) every day for 3 weeks, while mice in the control group received equal volumes of saline. In siZyxin group, 3 mg of lentiviral vectors expressing shRNA targeting mouse Zyxin (Targeting Site: CGCUGGAUAAGAACUUUCA tt UGAAAGUUCUUAUCCAGCG tt) were injected into the tail vein every three days on the seventh day after bleomycin instillation, while the non-targeted control mock lentivirus was injected in control and BLM group. All mice were euthanized 3 weeks after BLM administration. Skin tissues were obtained for further experiments. All animal experiments were performed in accordance with the NIH Guide for the Care and Use of Laboratory Animals, with the approval of the Scientific Investigation Board of School of Life Sciences, Fudan University (2019-JS-011).

### Human skin explant culture

Samples were delivered to the laboratory within 12 h of surgery on ice. The skin explants with an average diameter of 10 mm were cultured at 37ºC and 5% CO_2_ in DMEM containing 10% fetal bovine serum (FBS), and then transfected with Zyxin siRNA (20 nM) or control siRNA (20 nM) using Lipofectamine RNAiMAX (Invitrogen, USA). Those siRNAs were injected subcutaneously or added directly to the medium. The siRNA for negative control (NC) and Zyxin were purchased (GenePharma, Shanghai, China) and diluted in RNase-free double distilled water. Sequences for Zyxin siRNA are as follows: Zyxin-siRNA forward, 5'-GTCCTCACACTTGTAACAC-3' and reverse, 5'-GTGTTACAAGTGTGAGGAC-3'. After 72 h, the explants were collected for further experiments.

### Establishment of keloid xenograft nude mouse model

The keloid xenograft nude mouse model was performed on nude mice of 7 weeks. First, the keloid tissues were cut into approximately 0.5 × 0.5 × 0.8 cm^3^ sections, and the weight was 200 mg per tissue block. Nude mice were then anesthetized and implanted with the dermal tissues of keloid on the dorsal skins. After 14 days of implantation, the mice were assigned into 2 groups for Zyxin siRNA or control siRNA injection. For each transplant, siRNA (GenePharma, Shanghai, China, 5' Cholesterol modification) was dissolved into 50 μl PBS at a concentration of 2 nmol/20g and injected into keloid xenograft 2-3 times a week for four weeks. The weight of the keloids was determined at the time of excision after 4 weeks and the xenografts were harvested for further analysis.

### Cell culture and treatment

The isolated Human skin fibroblasts were cultured in Dulbecco's Modified Eagle's Medium (DMEM) (Invitrogen, CA, USA) with 10% FBS at 37 °C in a 5% CO2 humidified incubator. For RNA interference experiment, cells were transfected with Zyxin siRNA (20 nM) or control siRNA (20 nM) by adding the siRNA into the medium as described in previous method in the 5th section. For the over-expression experiment, lentivirus plasmids were constructed by Genomeditech (Shanghai, China). Cells were transduced with pLVX-mCherry-N1 according to the manufacturer's instructions. After 24 h, puromycin (Solarbio, Beijing, China) was used to confirm transfection efficiency. Stably transfected cells were obtained for further experiment. For the western blot experiment, protein levels of phosphorylated and total FAK, PI3K, or AKT were assessed in fibroblasts following treatment with recombinant TGF-β protein (HY-P70543, MCE, USA, 10 ng/ml) for 6 hours, with or without pretreatment with Zyxin siRNA. Protein levels of phosphorylated and total Smad2/3, ERK, JNK, or p38 were assessed in fibroblasts following treatment with TGF-β for 2 hours, with or without pretreatment with Zyxin siRNA. For the pan integrin antagonist GLPG0187 treated experiment, fibroblasts were pre-treated with GLPG0187 for 24 hours.

### RNA sequencing and data analysis

Total RNA extracted from SSc fibroblasts or siZyxin-treated SSc fibroblasts were used for library preparation and sequenced on a Genome Analyzer Hiseq 2500 (Illumina, San Diego, CA, USA). FastqC was used to investigate the sequence quality and FASTX_Toolkit was used to filter the low-quality reads. The data was analyzed by TopHat2 (version 2.1.0). Cufflinks (version 2.2.1) were performed to calculate the expression values and determine differentially expressed genes. Fragments per kilobase of exon per million fragments mapped (FPKM) was calculated as a relative expression level. The R package clusterProfiler (version 3.8.1) was used to conduct enrichment analysis of DEGs (q-value < 0.05), GO functional annotation, and the KEGG metabolic pathways. R package ggplot2 (version 3.0.0) was used to draw the plots.

### 3D spheroid model

For 3D spheroids formation, 0.5 × 10^4^, 1.0 × 10^4^, and 2.0 × 10^4^ cells were diluted in 200 µL of culture medium without FBS respectively and seeded in 96-well plates previously coated with 50 µL of 2% low-melting agarose at room temperature. The spheroid was formed with SSc primary fibroblasts treated with or without Zyxin siRNA. After shaking the plates gently, the plate was incubated at 37°C and 5% CO_2_. The images of spheroids were captured at 0, 2, 6, 24, 48 h after treatment by a microscope (Olympus, Japan).

### Migration and Invasion Assay

For the scratch wound assay, cells with or without transfection were plated into 12-well plates. After reaching 100% confluence, cells were treated with 2 μg/mL Mitomycin C (M5353, Sigma) for 1 h. The cells were then washed with PBS and wounded by scraping with a 200 μl pipette tip. After washing with PBS for 3 times, the cells were then incubated in DMEM without FBS. Photographs of the wound area were taken at 0, 24, 48, and 72 h.

### Collagen measurements and western blot analysis

Soluble collagen in skin samples was measured by the Sircol colorimetric assay (Biocolor, Belfast, UK) according to the manufacturer's protocol. The amount of collagen protein in skin samples was normalized to the total amount of protein as determined using a BCA Protein Assay kit (Thermo Fisher Scientific Inc, USA). Finally, the amount of solubilized collagen in each sample were expressed as the fold change compared to that of each control group. Intracellular proteins were extracted from cells by ice-cold RIPA lysis buffer (P0013, Beyotime) with 1mM phenylmethylsulfonyl fluoride (PMSF) (Thermo Scientific, USA). Equal amounts of proteins from each sample were loaded onto 10% SDS-polyacrylamide gels and transferred onto polyvinylidene difluoride (PVDF) membranes (Millipore, Billerica, MA, USA). After blocking with 5% BSA (servicebio, China), blotted proteins were incubated with antibodies at 4℃ overnight. The antibodies are as follows: pPI3K (1:1000, Cell Signaling Technology, USA), PI3K (1:1000, Cell Signaling Technology, USA), pAKT (1:2000, Cell Signaling Technology, USA), AKT (1:1000, Cell Signaling Technology, USA), pFAK (1:1000, Cell Signaling Technology, USA), pFAK(1:1000, Cell Signaling Technology, USA), Zyxin (1:10000, Abcam, UK). Then, the membranes were washed 3 times with TBST for 10 min each time and incubated with HRP-conjugated secondary rabbit antibodies (servicebio, China) or HRP-conjugated secondary mouse antibodies (servicebio, China) for 2 h. Finally, protein bands were re-washed with TBST and visualized with an enhanced chemiluminescence system (Thermo Fisher Scientific Inc., USA). The intensity of bands was quantified using Image-QuantTL software (General Electric Company, USA).

### Statistical analysis

All data are presented as the mean ± SD, and group differences were tested for statistical significance using an independent two-group t-test or one-way ANOVA test. P values < 0.05 were considered significant.

## Supplementary Material

Supplementary figures and tables.

## Abbreviations

SSc: systemic sclerosis
ECM: extracellular matrix
FA: focal adhesion
FAK: focal adhesion kinase
PI3K: phosphatidylinositol 3 kinase
AKT: protein kina
GEO: Gene Expression Omnibus
DEGs: differentially expressed genes
BLM: bleomycin
HFF-1: fibroblast cell line
FPKM: Fragments Per Kilobase per Million
