# Supplementary material for: Enhancement of Zyxin Promotes Skin Fibrosis by Regulating FAK/PI3K/AKT and TGF-β Signaling Pathways via Integrins
Source: Int J Biol Sci. 2023 Apr 29;19(8):2394–408. doi: 10.7150/ijbs.77649 (PMC10197900; doi:10.7150/ijbs.77649)
Supplement: Supplementary file 1 — Supplementary figures and tables. [file ijbsv19p2394s1.pdf]

Supplementary figures and figure legends

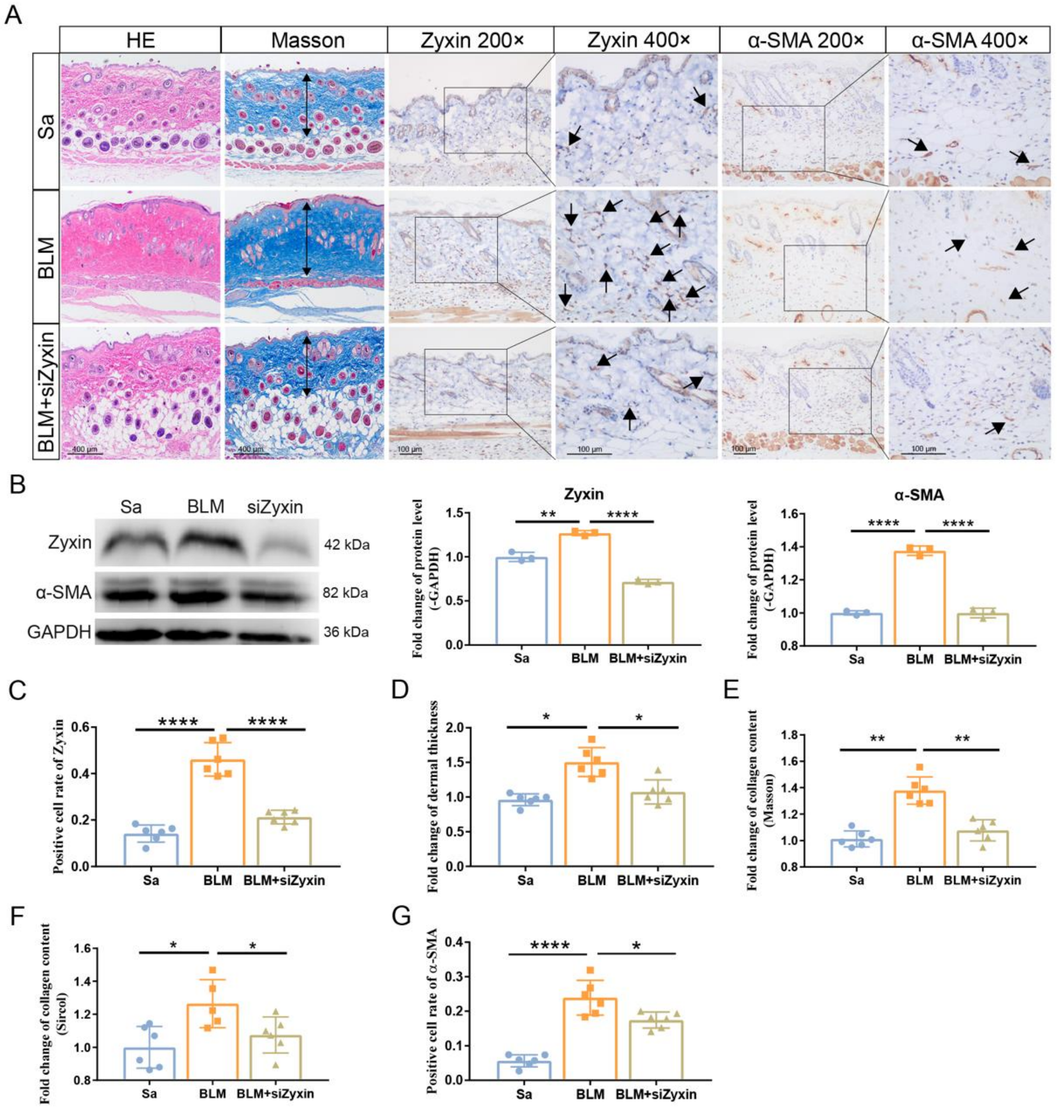

Figure S1. Knockdown Zyxin alleviated skin fibrosis in BLM-induced model mice. (A) Sections were stained with IHC, HE, and Masson's trichrome in different treatment groups. The arrow indicated Zyxin-positive cells. (B) Relative protein levels of α-SMA and Zyxin in mouse skin were measured by Western blotting. (C) The positive cell rate of Zyxin in different treatment groups. (D) Dermal thickness of mouse skin in different groups was calculated. (E, F) Collagen content of mouse skin in different groups was measured by ImageJ and Sircol collagen kit. (G) The positive cell rate of α-SMA in different treatment groups. Data are presented as mean±SD in two groups and compared by *t*-test. **P<0.05, **P*<0.01*, ***P*<0.001*, ****P*<0.0001.

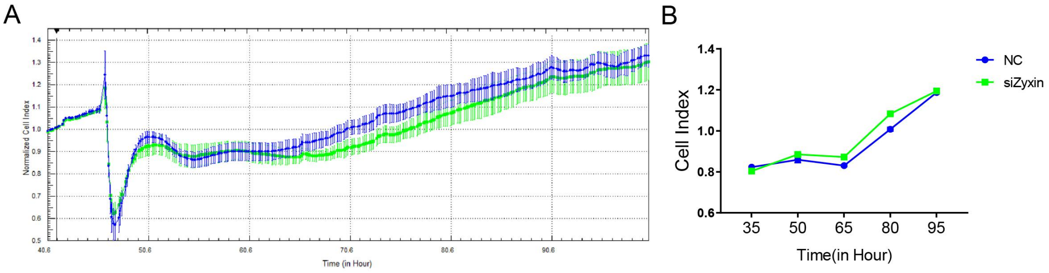

Figure S2. Zyxin does not affect the proliferative ability of fibroblasts. (A) Growth curves (measured as Cell Indices over time) of SSc skin fibroblasts treated with Zyxin siRNA or not were measured with the xCELLigence system. (B) Quantitative results of cell index. Data are presented as means±SD of the group and compared by *t*-test.

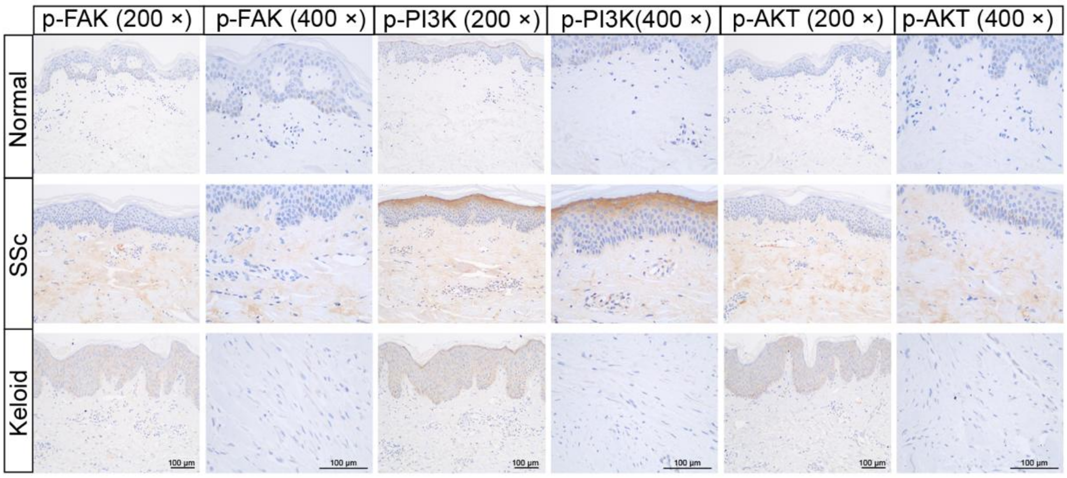

Figure S3. Focal adhesion signaling pathway was up-regulated in the fibrotic skin of SSc. Sections were stained with immunohistochemistry staining of the p-FAK, p-PI3K, and p-AKT in normal, SSc, and keloid patients.

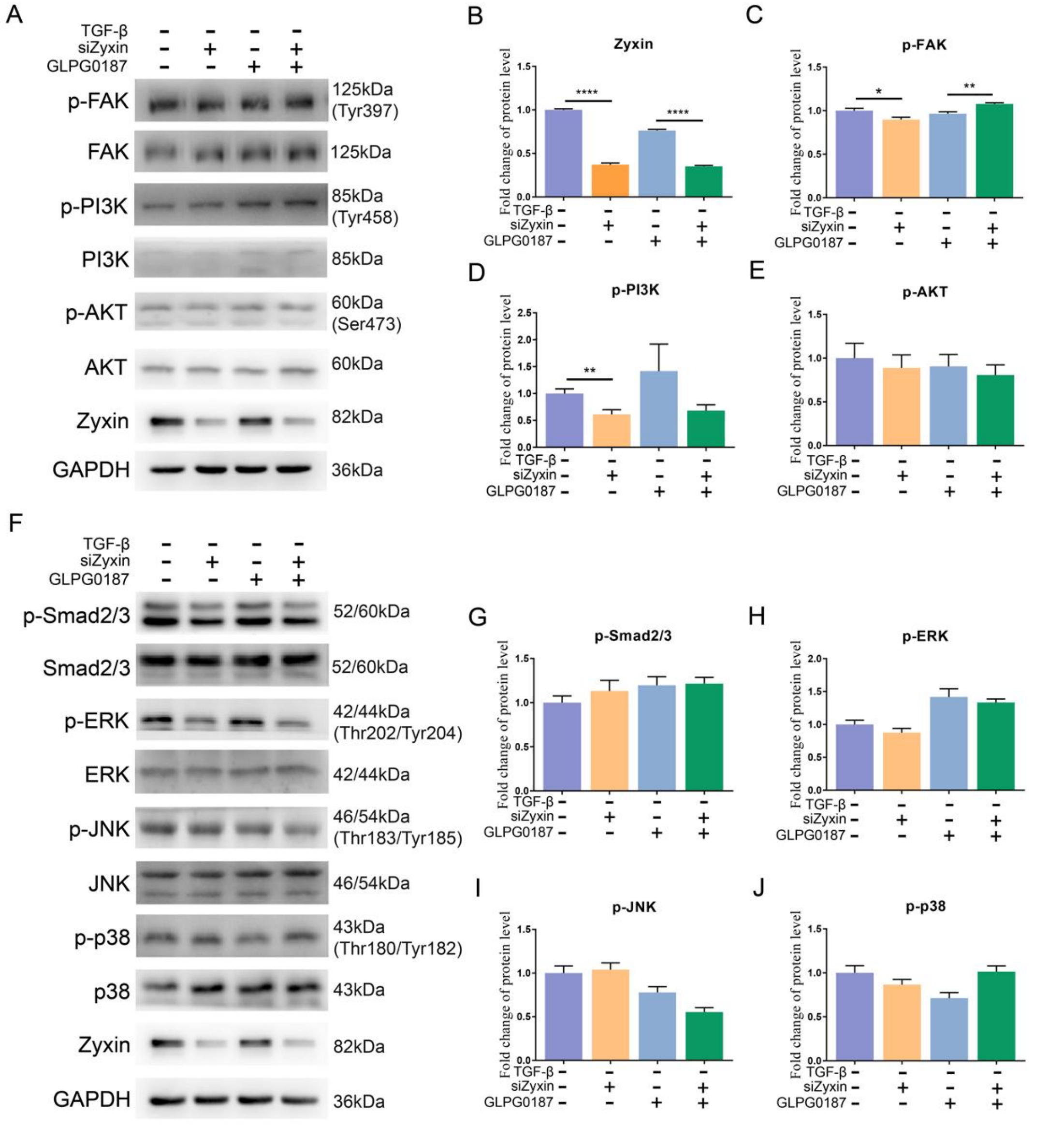

Figure S4. The role of integrins in mediating focal adhesion and TGF-β signaling pathways regulated by Zyxin in fibroblasts without treatment of TGF-β. (A) Levels of phosphorylated and total FAK, PI3K, or AKT were assessed in fibroblasts by Western blotting. In addition, fibroblasts with or without treatment with Zyxin siRNA were pre-treated with or without GLPG0187 for 24 hours. (B-E) Semi-quantification of the Western blot results by ImageJ. (F) Levels of phosphorylated and total Smad2/3, ERK, JNK, or p38 were assessed in fibroblasts by Western blotting. In addition, fibroblasts with or without treatment with Zyxin siRNA were pre-treated with or without GLPG0187 for 24 hours. (G-J) Semi-quantification of the Western blot results by ImageJ. Data are presented as means±SD in two groups and compared by *t*-test. **P*<0.05, ***P*<0.01, *****P*<0.0001.

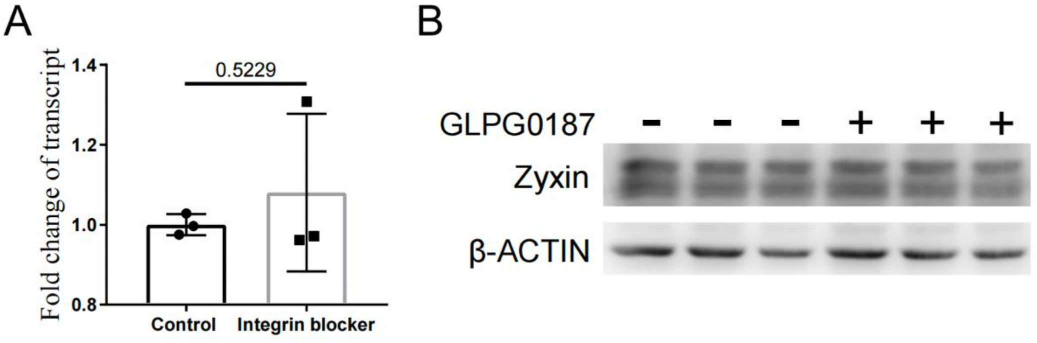

Figure S5. The effects of integrin blockers on Zyxin expression. (A) The mRNA expression level of *Zyxin* after integrin blocker GLPG0187 treatment in HFF-1 cells. (B) The relative protein level of Zyxin in cultured HFF-1 cells treated with or without integrin blocker GLPG0187.

Table S1. The list of DEGs in focal adhesion pathway.

Gene	baseMean	log2FC	lfcSE	stat	pvalue	padj
NOX4	3.93	2.06	0.23	8.76	1.88E-18	4.63E-16
THY1	46.34	1.66	0.15	11.35	7.55E-30	1.52E-26
TNC	58.65	1.62	0.16	10.03	1.09E-23	5.86E-21
FAP	18.86	1.45	0.16	9.15	5.51E-20	1.83E-17
MME	9.22	1.38	0.15	9.01	2.14E-19	6.15E-17
TGM2	2.07	1.37	0.27	5.00	5.73E-07	1.81E-05
ITGB2	18.15	1.02	0.12	8.39	5.04E-17	9.82E-15
SCARF2	5.34	0.92	0.16	5.94	2.86E-09	1.58E-07
ICAM1	10.47	0.90	0.13	7.03	2.11E-12	2.07E-10
APBB1IP	4.08	0.87	0.18	4.96	7.17E-07	2.20E-05
ITGA4	8.59	0.84	0.12	7.00	2.62E-12	2.52E-10
AKAP12	14.62	0.84	0.10	8.61	7.01E-18	1.59E-15
ITGBL1	39.35	0.84	0.13	6.61	3.87E-11	3.08E-09
DAB2	61.39	0.83	0.09	9.26	2.01E-20	7.30E-18
HCK	7.77	0.78	0.13	6.20	5.69E-10	3.66E-08
CYBA	36.88	0.77	0.10	8.04	8.91E-16	1.41E-13
ENG	14.85	0.74	0.12	6.16	7.24E-10	4.54E-08
ARPC1B	45.00	0.72	0.08	8.86	8.23E-19	2.11E-16
PTPRC	10.11	0.71	0.13	5.52	3.39E-08	1.48E-06
CNN3	66.33	0.67	0.07	9.59	8.50E-22	3.78E-19
NEDD9	15.06	0.66	0.09	7.45	9.26E-14	1.11E-11
NRP1	56.89	0.63	0.06	10.33	5.16E-25	3.69E-22
MCAM	47.85	0.58	0.11	5.18	2.24E-07	7.80E-06
FERMT2	20.91	0.55	0.08	7.18	6.83E-13	7.28E-11
TNS3	28.87	0.55	0.07	7.46	8.34E-14	1.01E-11
MMP14	62.86	0.54	0.07	7.18	7.23E-13	7.68E-11
LCP1	40.02	0.52	0.10	5.10	3.41E-07	1.12E-05
ITGA1	18.94	0.51	0.08	6.36	1.97E-10	1.37E-08
CNN2	35.13	0.51	0.08	6.06	1.37E-09	8.13E-08
LRP1	112.34	0.50	0.10	5.17	2.40E-07	8.23E-06
ZYX	21.44	0.50	0.11	4.70	2.65E-06	7.04E-05
LAP3	34.89	0.49	0.08	6.09	1.10E-09	6.63E-08
FAT1	59.23	0.47	0.08	6.08	1.17E-09	7.00E-08
ACTN1	62.85	0.46	0.08	5.53	3.25E-08	1.42E-06
ANXA6	52.04	0.45	0.06	7.57	3.62E-14	4.56E-12
MARCKS	32.94	0.43	0.06	7.01	2.33E-12	2.26E-10
MAP4K4	35.97	0.42	0.06	7.55	4.28E-14	5.37E-12

**PARVA**	22.51	0.41	0.07	5.76	8.48E-09	4.22E-07
**PEAK1**	36.68	0.41	0.06	6.93	4.08E-12	3.74E-10
**MYH9**	134.90	0.38	0.06	6.04	1.50E-09	8.85E-08
**ITGB1**	95.49	0.38	0.05	7.71	1.24E-14	1.67E-12
**TPM4**	107.52	0.37	0.06	6.50	7.88E-11	5.92E-09
**PPIB**	114.52	0.37	0.05	7.16	8.13E-13	8.56E-11
**MSN**	50.57	0.36	0.05	6.91	4.72E-12	4.23E-10
**ANXA5**	116.77	0.35	0.06	5.52	3.39E-08	1.48E-06
**ACTB**	863.92	0.34	0.07	4.67	3.02E-06	7.87E-05
**PALLD**	82.50	0.33	0.06	5.17	2.30E-07	7.95E-06
**ENAH**	34.13	0.32	0.07	4.93	8.28E-07	2.48E-05
**EPB41L2**	44.80	0.32	0.06	5.25	1.53E-07	5.63E-06
**TLN1**	59.55	0.31	0.07	4.72	2.36E-06	6.34E-05
**CAV1**	87.21	0.28	0.06	4.94	7.68E-07	2.33E-05
**VCL**	48.73	0.27	0.05	5.67	1.46E-08	6.92E-07
**CD151**	68.15	0.26	0.05	5.34	9.32E-08	3.60E-06
**CALR**	125.33	0.25	0.05	5.62	1.95E-08	8.93E-07
**CFL1**	187.53	0.25	0.04	6.00	1.97E-09	1.11E-07
**SDCBP**	57.90	0.24	0.05	4.93	8.19E-07	2.46E-05
**ARPC5**	67.68	0.23	0.04	5.65	1.63E-08	7.66E-07
**PFN1**	122.52	0.22	0.05	4.79	1.66E-06	4.59E-05
**HSP90B1**	96.84	0.19	0.04	5.51	3.50E-08	1.52E-06
**CAP1**	78.42	0.19	0.04	4.91	9.27E-07	2.70E-05
**CTNNB1**	136.34	0.17	0.03	4.75	2.03E-06	5.51E-05
**RPL18**	759.51	-0.20	0.04	-4.84	1.32E-06	3.71E-05
**RPL38**	597.31	-0.23	0.05	-4.84	1.27E-06	3.59E-05
**RPL5**	213.12	-0.23	0.04	-5.25	1.53E-07	5.63E-06
**RPL31**	408.23	-0.24	0.05	-4.70	2.60E-06	6.93E-05
**RPS14**	579.83	-0.24	0.05	-4.95	7.41E-07	2.26E-05
**RPL30**	733.63	-0.24	0.05	-4.97	6.80E-07	2.10E-05
**EZR**	73.95	-0.25	0.05	-4.66	3.15E-06	8.14E-05
**RPS10**	66.43	-0.25	0.05	-4.95	7.41E-07	2.26E-05
**AHNAK**	622.64	-0.26	0.03	-8.49	2.02E-17	4.29E-15
**SVIL**	74.67	-0.27	0.05	-5.76	8.65E-09	4.29E-07
**RPLP1**	442.57	-0.28	0.06	-4.92	8.68E-07	2.58E-05
**RPS7**	44.06	-0.28	0.05	-5.83	5.65E-09	2.92E-07
**CD9**	150.12	-0.31	0.05	-6.23	4.54E-10	2.95E-08
**AJUBA**	28.20	-0.38	0.07	-5.86	4.72E-09	2.49E-07
**GSN**	440.99	-0.67	0.09	-7.54	4.54E-14	5.66E-12

The normal group was used as the reference group; log2FC, log2 fold change.

Table S2. The list of differentially expressed of integrins.

Gene	mean_obs	log2FC	stat	pvalue	qvalue
ITGA1	3.46	-1.12	8.46	0.003632416	0.108764957
ITGA11	1.31	-2.62	5.51	0.018953645	0.277421469
ITGA2	7.06	-0.29	10.43	0.001238824	0.058295827
ITGA3	7.72	-0.39	15.83	6.93E-05	0.010550958
ITGAV	1.49	-2.68	4.72	0.029799625	0.345729548
ITGB1	10.35	0.24	5.17	0.022937881	0.303652912
ITGB2	1.14	1.19	0.98	0.323146196	0.841118271
ITGB3	6.01	-0.53	12.94	0.000321983	0.025486835
ITGB5	8.03	-0.54	12.58	0.000389909	0.028369543
ITGB8	5.94	0.83	19.12	1.23E-05	0.004543678
ITGBL1	6.63	-0.59	16.32	5.34E-05	0.009408206

The NC group was used as the reference group; log2FC, log2 fold change.

Table S3. The list of real-time RT-PCR primers.

Primer Name	Species	Sequence (5' to 3')
Zyxin-F	human	CGGCTCAGAACCAAAACCAG
Zyxin-R	human	CAGAGTTCGTTGACAGCCACATT
α-SMA-F	human	GAGCGTGGCTATTCCTTCGT
α-SMA-R	human	GCCCATCAGGCAACTCGTAA
ITGA1-F	human	AATTGGCTCTAGTCACCATTGTT
ITGA1-R	human	CAAATGAAGCTGCTGACTGGT
ITGA3-F	human	GAGGACATGTGGCTTGGAGT
ITGA3-R	human	GTAGCGGTGGGCACAGAC
ITGA11-F	human	CTTTTCCTCGCACGTGGT
ITGA11-R	human	GCTCCATTCCAGTCATAGGC
ITGB3-F	human	CATCCACGACCGAAAAGAA
ITGB3-R	human	TGAAGGTAGACGTGGCCTCT
ITGB5-F	human	GGAGTTTGCAAAGTTTCAGAGC
ITGB5-R	human	TGTGCGTGGAGATAGGCTTT
COL1A1-F	human	CATCTGGTGGTGAGACTTGC
COL1A1-R	human	TCCTGGTTTCTCCTTTGG
COL1A2-F	human	AAGGTCATGCTGGTCTTGCT
COL1A2-R	human	GACCCTGTTCACCTTTTCCA
COL1A3-F	human	GTCCCAGCGGTTCTCCA
COL1A3-R	human	CCCCGTGCTCCAGTGAT
FN1-F	human	CGGTGGCTGTCAGTCAAAG
FN1-R	human	AAACCTCGGCTTCCTCCATAA
CTGF-F	human	GGAAATGCTGCGAGGAGTGG
CTGF-R	human	GGCTCTAATCATAGTTGGGTCTGG
PAI-F	human	AGTGGACTTTTCAGAGGTGGAG
PAI-R	human	GCCGTTGAAGTAGAGGGCATT
β-ACTIN-F	human	AGCGCGGCTACAGCT
β-ACTIN-R	human	GGCCATCTCTTGCTCGAAGT

F, Forward primer; R, Reverse primer.
